# Supplementary material for: Dynamic transcriptomic profiles of zebrafish gills in response to zinc supplementation
Source: BMC Genomics. 2010 Oct 11;11:553. doi: 10.1186/1471-2164-11-553 (PMC3091702; doi:10.1186/1471-2164-11-553)
Supplement: Additional file 2 — Interactive Direct Interaction Network representing the molecular interactions between zinc, copper, iron, calcium and proteins encoded by transcripts changed by zinc supplementation. Mini web-site containing index.html and hyperlinked pages in subdirectory describing a Direct Interaction Network automatically generated based on curated interactions contained within the proprietary PathwayArchitect database. Ovals represent proteins and the circles symbolize metal ions. Objects are coloured by their abundance in zebrafish at the time-point they were significantly different from the control is a scale from -4 fold (dark green) to +4 fold (dark red). Where significant differences were found at more than one time-point, the colour overlay shows expression at the first instance. Dark blue squares denote 'binding', and light blue squares 'expression'; green squares stand for 'regulation', green diamonds for 'metabolism', and green circles for 'promoter binding'. Arrow heads indicate directionality of the interaction where annotated. All nodes and edges can be further interrogated by selecting the relative area of the image. [file 1471-2164-11-553-S2.zip › PathwayArchitect Zn xs DIN/76942.html]

# PROTEIN: PAX9

|  |  |
| --- | --- |
| Name | PAX9 |
| Type | PROTEIN |
| Description | paired box gene 9 |
| Note | This gene is a member of the paired box (PAX) family of transcription factors. Members of this gene family typically contain a paired box domain, an octapeptide, and a paired-type homeodomain. These genes play critical roles during fetal development and cancer growth. The specific function of the paired box gene 9 is unknown but it may involve development of stratified squamous epithelia as well as various organs and skeletal elements. |
| Alias | Paired box protein Pax-9 |
|  | paired domain gene 9 |
|  | paired box 9 |
|  | Pax-9 |
|  | PAX9 |
|  | Pax9 |


---

|  |  |
| --- | --- |
| GO Component | transcription factor complex |
|  | nucleus |


---

|  |  |
| --- | --- |
| GO ID | GO:0007492 |
|  | GO:0005634 |
|  | GO:0003677 |
|  | GO:0006355 |
|  | GO:0009887 |
|  | GO:0007275 |
|  | GO:0005667 |
|  | GO:0006350 |
|  | GO:0003700 |


---

|  |  |
| --- | --- |
| MIM | MIM:106600 |
|  | MIM:167416 |
|  | MIM:604625 |


---

|  |  |
| --- | --- |
| Connectivity | 24 |


---

|  |  |
| --- | --- |
| Entrez ID | 5083 |
|  | 18511 |


---

|  |  |
| --- | --- |
| Agilent ID | A\_52\_P593379 |
|  | A\_32\_P70818 |
|  | A\_32\_P70811 |
|  | A\_53\_P163625 |
|  | A\_14\_P124552 |
|  | A\_14\_P115497 |
|  | A\_51\_P214602 |
|  | A\_14\_P202209 |
|  | A\_23\_P426944 |
|  | A\_53\_P156804 |


---

|  |  |
| --- | --- |
| Cellular Localization | Nucleus |
|  | Organelle |
|  | Cell |


---

|  |  |
| --- | --- |
| Pathway | Zn xs inventory |
|  | Zn xs DIN |


---

|  |  |
| --- | --- |
| GO Process | development |
|  | regulation of transcription, DNA-dependent |
|  | organ morphogenesis |
|  | endoderm development |
|  | transcription |


---

|  |  |
| --- | --- |
| UniGene | Hs.132576 |
|  | Mm.5035 |


---

|  |  |
| --- | --- |
| Affymetrix Probeset ID | 231145\_at |
|  | 1421246\_at |
|  | 1421247\_at |
|  | 207059\_at |
|  | 1442229\_at |
|  | 34933\_at |
|  | 73402\_at |
|  | 98838\_at |
|  | g7242166\_3p\_at |
|  | X84000\_s\_at |
|  | 64709\_at |
|  | Hs.55080.0.A1\_3p\_at |
|  | RC\_W37683\_at |


---

|  |  |
| --- | --- |
| GO Function | transcription factor activity |
|  | DNA binding |


---

|  |  |
| --- | --- |
| Nucleotide | AJ238383 |
|  | BC005794 |
|  | X92850 |
|  | AY338688 |
|  | X73037 |
|  | U59628 |
|  | AJ238382 |
|  | AK132404 |
|  | L09745 |
|  | AK034985 |
|  | NM\_011041 |
|  | BC001159 |
|  | AJ238381 |
|  | NM\_006194 |
|  | X84000 |
|  | BC006930 |


---

|  |  |
| --- | --- |
| Protein | AAQ16120 |
|  | NP\_006185 |
|  | BAC28903 |
|  | P55771 |
|  | AAH05794 |
|  | P47242 |
|  | AAD09487 |
|  | CAA51518 |
|  | NP\_035171 |
|  | CAA58824 |
|  | CAB41533 |
|  | AAH01159 |
|  | BAE21149 |
|  | CAA63436 |


---

|  |  |
| --- | --- |
| Organism | Mammal |


---

|  |  |
| --- | --- |
| Location | chromosome 12, 12 26.0 cM, 12 C1 (Mus musculus) |
|  | 12 26.0 cM (Mus musculus) |
|  | chromosome 14, 14q12-q13 (Homo sapiens) |


---

|  |  |
| --- | --- |
